# Comparing Combined Non-ablative Fractional Laser and Radiofrequency Microneedling Versus Non-ablative Fractional Laser Alone for Acne Scar Treatment: A Meta-Analysis

**DOI:** 10.7759/cureus.99900

**Published:** 2025-12-22

**Authors:** Hosam Hadi Hassan Awaji, Basel H Bakhamees, Mohammed Alruwaili, Faris Alhumaid, Saja A Albenayyan, Abdulkareem A Ajaj, Wejdan M Alzahrani, Abdulla Jan, Saud Almutiri, Somia M Alshafie, Sami S Alghamdi, Abdulrahman O Alfakhri, Fatimah S Alhajri

**Affiliations:** 1 Preventive Medicine, North West Armed Forces Hospitals, Tabuk, SAU; 2 Medicine, King Abdulaziz University, Jeddah, SAU; 3 Surgery, King Salman Armed Forces Hospital, Tabuk, SAU; 4 Surgery, Qassim University, Buraydah, SAU; 5 College of Medicine, Imam Abdulrahman Bin Faisal University, Dammam, SAU; 6 Medicine, Ibn Sina National College for Medical Studies, Jeddah, SAU; 7 College of Medicine, Umm Al-Qura University, Mecca, SAU; 8 General Practice, University of Jeddah, Jeddah, SAU; 9 Dermatopathology, King AbdulAziz Specialist Hospital, Sakaka, SAU; 10 Gynecology, Suez Canal University Hospital, Ismailia, EGY; 11 General Medicine, King Fahad Hospital, Al Bahah, SAU; 12 General Practice, University of Tabuk, Tabuk, SAU; 13 Dermatology, Nottingham Trent University, Nottingham, GBR

**Keywords:** acne, acne scar treatment, fractional microneedling radiofrequency, non-ablative fractional laser, scars

## Abstract

The combination of fractional microneedling radiofrequency (FMR) and fractional laser (FL) is a popular multimodal treatment for atrophic acne scars, yet its superiority over FL monotherapy remains uncertain based on conflicting primary studies. This meta-analysis aimed to synthesize evidence from randomized split-face trials comparing the efficacy of combined FMR+FL versus FL alone. A systematic search identified five eligible studies comprising 116 patients. Pooled odds ratios (ORs) and mean differences (MDs) with 95% confidence intervals (CIs) were calculated for primary outcomes, including achievement of 50-75% improvement, change in the Échelle d’Évaluation Clinique des Cicatrices d’Acné (ECCA), also known as the Acne Scar Grading Scale, and patient-assessed global improvement. The overall analysis showed no significant advantage for combination therapy across all outcomes (improvement OR: 1.22, 95% CI: 0.70-2.12, p=0.48; ECCA MD: -6.47, 95% CI: -21.68-8.73, p=0.40; Global improvement MD: 0.36, 95% CI: -0.22-0.94, p=0.22). Substantial heterogeneity was noted for ECCA (I^2^=91%) and patient improvement (I^2^=83%). Subgroup analysis indicated a borderline significant benefit for the FMR+ablative fractional laser (AFL) combination (OR: 2.76, 95% CI: 0.99-7.71, p=0.05) but not for FMR+non-ablative fractional laser (NAFL). Current evidence does not conclusively support the routine use of combined FMR+FL over FL monotherapy for atrophic acne scars. A potential benefit may exist specifically when combining FMR with AFL, warranting further investigation in larger, standardized trials.

## Introduction and background

Atrophic acne scarring is a prevalent and psychologically burdensome sequela of acne vulgaris, affecting a significant portion of the global population and often persisting as a lifelong concern. The psychosocial impact is substantial, with studies consistently linking facial scarring to reduced quality of life, social anxiety, and depression, driving a strong demand for effective treatments [1]. The search for optimal, evidence-based treatments remains a central challenge in dermatology, driven by the condition's complex pathophysiology involving dermal matrix damage and irregular collagen remodeling [2]. Among the myriad of interventions, fractional laser (FL) therapy, both ablative (AFL) and non-ablative (NAFL), has established itself as a cornerstone of treatment by creating controlled microthermal zones to stimulate neocollagenesis and skin resurfacing [3]. However, the quest for superior outcomes and the mechanistic appeal of simultaneously targeting different depths of dermal pathology have popularized the combination of FL with fractional microneedling radiofrequency (FMR), a modality that delivers focused thermal energy to the deep dermis to induce collagen contraction and tightening [4,5].

The theoretical synergy of this combination is compelling. FL addresses epidermal texture and superficial dermal remodeling, while FMR purports to provide deeper volumetric heating and collagen stimulation [6,7]. Clinically, this multimodal approach has been widely adopted with the assumption that it yields superior cosmetic improvement compared to laser monotherapy. However, the published evidence is fragmented, comprising small-scale, split-face studies with inconsistent and sometimes contradictory results. Some recent trials suggest a pronounced benefit, particularly with AFL combinations, while others report negligible additive value, raising critical questions about the universal applicability and cost-effectiveness of this increasingly common practice [7-9].

This inconsistency in the literature creates a significant dilemma for clinicians and patients navigating treatment choices. Without a synthesized, quantitative analysis, it remains unclear whether the added complexity, cost, and potential for increased adverse events of combination FMR+FL therapy are justified by a clinically meaningful improvement in outcomes [3]. Previous reviews have been limited in scope or are outdated, given the recent publication of pivotal trials [3,10]. Therefore, this meta-analysis was conducted to rigorously appraise and synthesize the current highest level of evidence from randomized split-face controlled trials. It aims to definitively compare the efficacy and patient-reported outcomes of combined FMR+FL therapy against FL monotherapy for atrophic acne scars, with prespecified subgroup analyses to explore the differential effects of AFL versus NAFL platforms, thereby providing evidence-based guidance for clinical practice.

## Review

Methods

The conduction and reporting of this meta-analysis followed the principles of the Cochrane Handbook for Systematic Reviews of Interventions, version 6, and the Preferred Reporting Items for Systematic Reviews and Meta-Analyses (PRISMA) guidelines [11].

The Research Question

Does the combination of NAFL and FMR provide superior efficacy and safety compared to NAFL alone in the treatment of acne scars?

Research Aim and Objective

The study aims to evaluate the comparative efficacy of combined NAFL and FMR versus NAFL alone in the treatment of acne scars, and the objective is to systematically review and synthesize existing studies that compare the efficacy of NAFL combined with FMR versus NAFL alone in reducing acne scar severity.

Inclusion Criteria

Types of studies: This meta-analysis included randomized controlled studies that were published from inception to 20 December 2025.

Participants: Eligible studies included all studies that compared the efficacy and adverse effects of a combination of NAFL and FMR versus NAFL alone in the treatment of acne scars. No restrictions were made with regard to age, sex, or race of the participants.

Interventions: Studies that were considered eligible included those that compared a combination of NAFL and FMR with NAFL alone in the treatment of acne scars.

Exclusion Criteria

We excluded studies involving animals, retrospective analyses, conference abstracts, duplicate entries, case reports, review articles, commentaries, case series with fewer than four patients, or clinical guidelines.

Search Strategy

Electronic searches: The following electronic databases were searched for eligible studies: MEDLINE/PubMed, Cochrane Central Register of Controlled Trials (CENTRAL), Web of Science, ProQuest, and Scopus. The search was set for all articles published in English.

The following search terms were used: (fractional microneedling radiofrequency) AND (fractional laser) AND (acne scars). We used no filters by language or publication period. The search terms used for each database and the count of search results were summarized in the Appendices.

Other resources: The first reviewer searched within the reference lists of obtained articles for other potentially relevant studies that were not retrieved by the electronic search.

Selection of Studies

The first reviewer screened the retrieved reports for eligibility through title and abstract, and full-text screening. The second reviewer checked the retrieved studies, and discrepancies were solved through discussion with a third reviewer.

Data Extraction

The first reviewer carried out data extraction from the included studies using a standardized data sheet that included: (a) the study’s characteristics (author, year, country, study design); (b) patients’ characteristics (age at the time of treatment, sex, sample size); (c) intervention details (type, dose, number of sessions, and the duration of follow up), and (e) the outcomes; achievement of 50-75% improvement in acne scars using the Échelle d’Évaluation Clinique des Cicatrices d’Acné (ECCA), also known as the Acne Scar Grading Scale [12], the mean subject-assessed global improvement scale score. The second reviewer checked the collected data for consistency and clarity. Any disagreements were settled by referring to the third reviewer.

Measured Outcomes

Outcomes included the proportion of participants achieving a 50-75% improvement in acne scars (events and total), the mean ± standard deviation (SD) scores on the ECCA, and the mean subject-assessed global improvement scale score.

Assessment of the Risk of Bias (ROB) in Included Studies

The ROB in the included studies was assessed using the National Institute for Health and Care Excellence (NICE) checklists for randomized controlled clinical trials [13].

Data Synthesis

**Figure 1:**
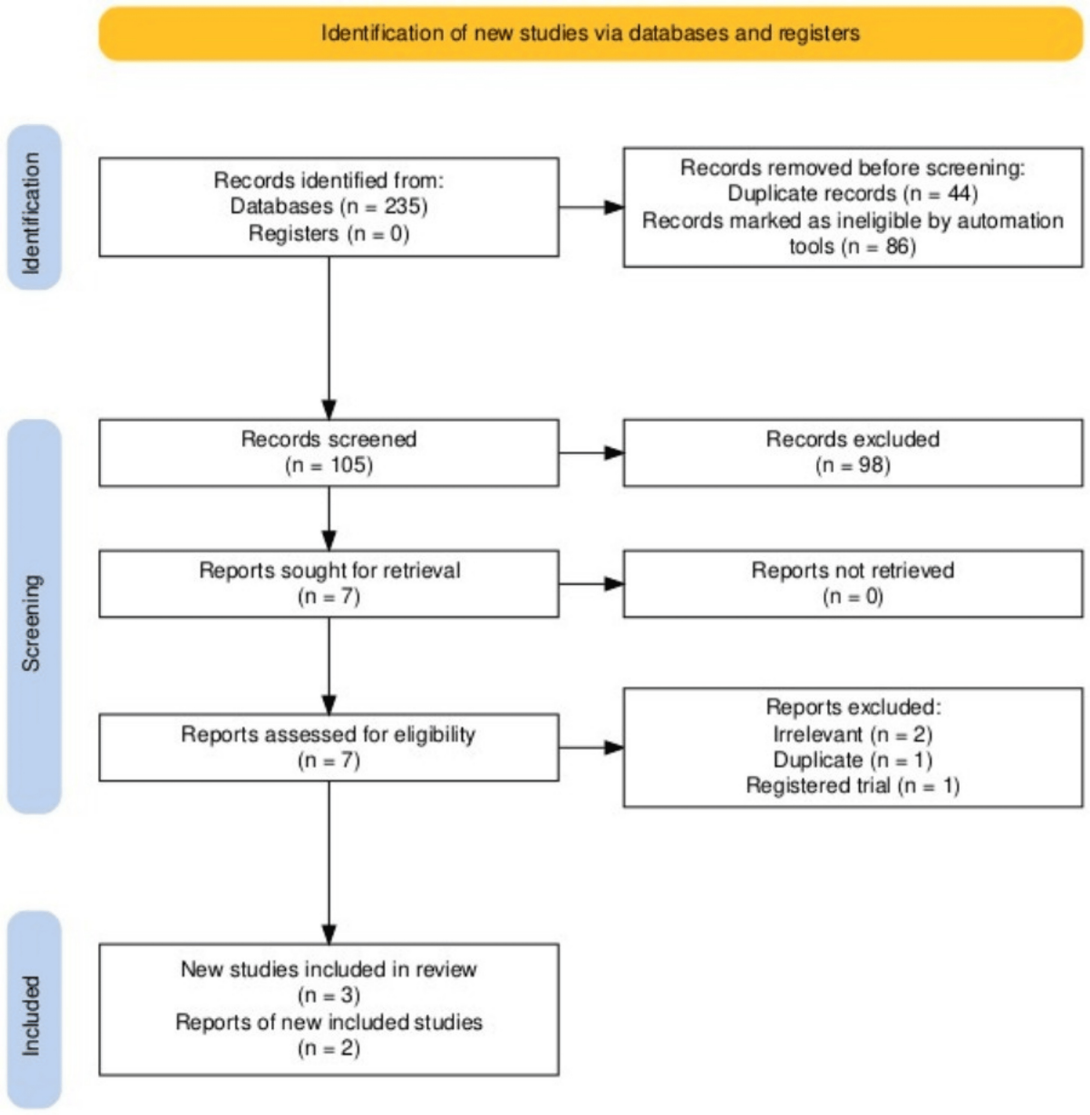
PRISMA flow chart PRISMA: Preferred Reporting Items for Systematic Reviews and Meta-Analyses

Initially, 235 records were retrieved from electronic database searches (see the Appendices). After removal of duplicates and screening of titles and abstracts, seven studies were assessed for eligibility. Of these, three studies met the inclusion criteria and were included in the meta-analysis [14-16]. Four studies were excluded because they were irrelevant (n=2), a registered trial without results (n=1), or a duplicate publication (n=1). Additionally, two articles were identified through grey literature searching [8,9]. Ultimately, five studies were included in the analysis, comprising a total of 116 patients and 232 facial sides (Table 1 and Figure 1) [17].

**Table 1:** Summary of the included studies FMR: fractional microneedling radiofrequency; AFL: ablative fractional laser; NAFL: non-ablative fractional laser; FRF: fractional radiofrequency

Author	Year	Country	Study design	Age		Sex (M: F)		Sample size		FMR+ FL (parameters, interval, and number of sessions)	FL (parameters, interval, and number of sessions)	Primary outcomes	Secondary outcomes	Side effects	Follow-up
				Combined	Laser alone	Combined	Laser alone	Combined	Laser alone						
Hartman et al. [14]	2024	USA	Prospective, randomized, split-face study	(25-44) 34.1 years (mean)	(25-44) 34.1 years (mean)	13:06	13:06	20 patients (split face)	20 patients (split face)	NAFL alternating with MNRF: 40-70 mJ, 14-20% coverage, eight passes, four sessions at four-week intervals	NAFL: 40-70 mJ, 14-20% coverage, eight passes, four sessions at four-week intervals	Improvement in atrophic acne scarring	Subject comfort, satisfaction, and adverse events	No significant difference between the groups	Three months after the last treatment
Kim et al. [15]	2023	Korea	Randomized, split face	24.1 ± 4.76	24.1 ± 4.76	16:07	16:07	23 (split face)	23 (split face)	Pulsed-wave mode (PW4), 1.6-2.0 mm microneedle depth, intensity level 4-6, one pass with <20% overlap, three sessions at four-week intervals	100 mJ, density of 100 spots/cm², 15.6% coverage, ablation depth of 1168 µm, three sessions at four-week intervals	Severity of inflammatory acne, acne scars, lesion counts, patient and physician satisfaction	Sebum output, skin biopsy findings	No significant difference between the groups	20 weeks
Kwon et al. [16]	2017	Korea	Prospective, randomized split-face study	21-38 years	21-38 years	15:13	15:13	26 (split face)	26 (split face)	1.5-2.5 mm microneedle depth, 20-50 intensity, 50-100 ms duration, three sessions at four-week intervals	25-35 J/cm², level 6, 4 passes, three sessions at four-week intervals	ECCA (Échelle d'évaluation clinique des cicatrices d'acné) score improvement	Histopathological and immunohistochemical results	No significant difference between the groups	Eight weeks after the final treatment
Kacar et al. [9]	2020	Turkey	Prospective, randomized, split-face, single-blinded, controlled	23.9 ± 4.4 years	23.9 ± 4.4 years	8:19	8:19	27 (split face)	27 (split face)	Sessions 1-3: FRF monthly. Session 4: Fractional CO₂ Laser (FCO₂). FRF Parameters: 25 semi-insulated microneedles, intensity 30-50%, duration 140-160 ms, delay 140-160 ms, depth 1.5-3 mm. FCO₂ Parameters (Session 4): Spot distance 1.3 mm (256 dots/20x20 mm, 0.87% coverage), energy 12-15 mJ, i-stack 2-4.	Sessions 1-4: FCO₂ monthly. FCO₂ Parameters: Spot distance 1.3 mm (256 dots/20x20 mm, 0.87% coverage), energy 12-15 mJ, i-stack 2-4.	Change in ECCA score	Patient-assessed improvement (5-point scale). Patient satisfaction (VAS 0-10).	Erythema, pain, and edema occurred on both sides. Side effects were longer-lasting on the FCO₂ side. Other effects: vesicles, erosions, petechiae, desquamation (infrequent). Pain scores (VAS) were higher for FCO₂ at Visits 1 and 3.	Six months
He et al. [8]	2024	China	Prospective, randomized, single-blind, split-face trial	Not stated	Not stated	Not stated	Not stated	20 (split face)	20 (split face)	Alternating FMR and AFL: FMR (sessions 1, 3) and AFL (sessions 2, 4) at four-week intervals.	AFL alone: Sessions 1-4 at four-week intervals.	ECCA score reduction	Sebum secretion reduction. Patient satisfaction. Procedural pain (VAS)	Transient erythema and crusting, comparable between groups.	24 weeks

Statistical Analysis

Meta-analysis was performed using Review Manager (RevMan) 5.4 version (The Cochrane Collaboration, London, England, UK) [18]. The outcome measures used in this meta-analysis included odds ratios (ORs), mean differences (MDs), and heterogeneity statistics. ORs were employed to assess the likelihood of achieving a 50-75% improvement in acne scars, providing a measure of the relative odds between the FMR+ FL and FL-alone groups. MDs were used to evaluate continuous outcomes, such as the ECCA and the subject-assessed global improvement scale, quantifying the difference in means between the two groups. Among the pooled studies with 95% confidence intervals (CIs) were calculated using a random-effects model when heterogeneity was expected among studies. Heterogeneity was assessed using the I^2^ statistic, with values above 50% indicating substantial heterogeneity. Sensitivity analyses were conducted by excluding studies one by one to assess the robustness of the results.

Results

After exclusion of irrelevant studies, five studies with a total number of 116 patients were included in this meta-analysis [8,9,14-16]. All the studies were right-to-left comparative studies comparing the efficacy of FMR+FL and FL alone in the treatment of post-acne atrophic scars.

Achievement of 50-75% improvement in Acne Scars

Five randomized controlled trials with a total of 116 patients were included in the meta-analysis comparing the achievement of 50-75% improvement in acne scars between FMR+FL versus FL alone [8,9,14-16]. The overall pooled OR was 1.22 (95% CI: 0.70 to 2.12, p=0.48), indicating no statistically significant difference between the two treatment modalities. There was low heterogeneity among the included studies (I^2^=23%).

**Figure 2:**
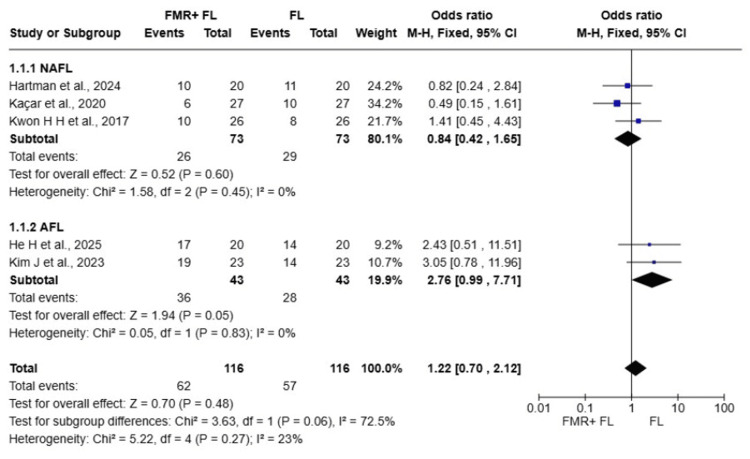
Forest plot comparing the number of facial sides achieving 50-75% improvement References [14,9,16,8,15] FMR: fractional microneedling radiofrequency; FL: fractional laser; AFL: ablative fractional laser; NAFL: non-ablative fractional laser; CI: confidence interval

Subgroup analysis revealed a trend toward differential treatment effects based on laser type (test for subgroup differences: p=0.06, I^2^=72.5%). In the NAFL subgroup (three studies, 73 patients), the pooled OR was 0.84 (95% CI: 0.42 to 1.65, p=0.60) with no heterogeneity (I^2^=0%). Conversely, in the AFL subgroup (two studies, 43 patients), the pooled OR was 2.76 (95% CI: 0.99 to 7.71, p=0.05) with no heterogeneity (I^2^=0%), suggesting a borderline significant benefit favoring the combination therapy in this subgroup (Figure 2).

Acne Grading Scar Scale (ECCA)

Four studies comprising 93 patients were pooled to compare the reduction in ECCA scores between FMR+FL and FL alone [8,9,14,16]. The overall random-effects meta-analysis showed a non-significant MD of -6.47 (95% CI: -21.68 to 8.73; p=0.40), indicating no clear advantage for the combined modality. Considerable heterogeneity was observed among the studies (I^2^=91%), which was not adequately resolved by sensitivity analysis or subgroup analysis.

**Figure 3:**
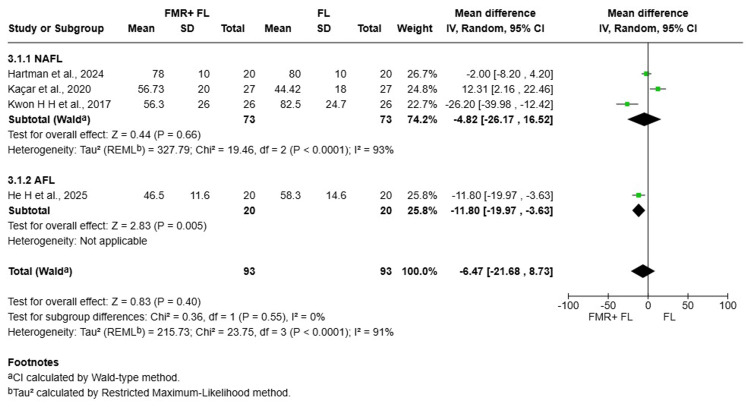
Forest plot comparing Acne Scar Grading Scale (ECCA) scores References [14,9,16,8] FMR: fractional microneedling radiofrequency; FL: fractional laser; AFL: ablative fractional laser; NAFL: non-ablative fractional laser; CI: confidence interval

Subgroup analysis by laser type revealed distinct patterns. In the NAFL subgroup (three studies, 73 patients), the pooled result was non-significant (MD -4.82, 95% CI: -26.17 to 16.52; p=0.66) and exhibited extreme heterogeneity (I^2^=93%). This high inconsistency stemmed from opposing effect directions: one study favored FL alone, while another favored the combination. In contrast, the single study in the AFL subgroup demonstrated a statistically significant reduction in ECCA scores favoring the combination therapy (MD -11.80, 95% CI: -19.97 to -3.63; p=0.005). However, the test for subgroup differences was not significant (I^2^=0%) (Figure 3).

The Mean Subject-Assessed Global Improvement Scale Score

Five randomized controlled trials with a total of 116 patients were pooled to compare patient-reported global improvement between FMR+FL and FL alone [8,9,14-16]. The overall random-effects meta-analysis yielded a non-significant MD of 0.36 (95% CI: -0.22 to 0.94; p=0.22), suggesting no statistically significant difference in subjective patient improvement between the combined and single-modality treatments. Substantial heterogeneity was observed among the included studies (I^2^=83%), which was not explained by subgroup analysis based on laser type (test for subgroup differences: p=0.89, I^2^=0%). But this heterogeneity was best resolved by the exclusion of Kwon et al. [16].

**Figure 4:**
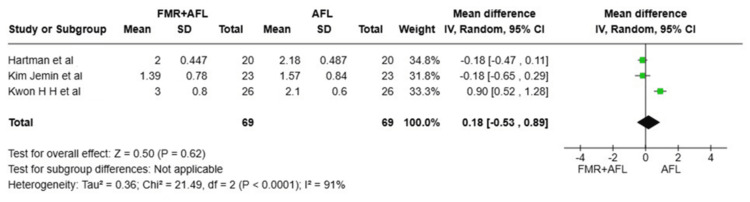
Forest plot comparing mean subject-assessed global improvement scale scores References [14-16] FMR: fractional microneedling radiofrequency; AFL: ablative fractional laser; CI: confidence interval

Subgroup analysis revealed no significant benefit for either subgroup. In the NAFL subgroup (three studies, 73 patients), the pooled MD was 0.38 (95% CI: -0.34 to 1.10; p=0.30) with considerable heterogeneity (I^2^=86%), driven by opposing effect directions among the studies. For the AFL subgroup (two studies, 43 patients), the pooled estimate was also non-significant (MD 0.51, 95% CI: -1.11 to 2.13; p=0.54) with high heterogeneity (I^2^=78%), where one study favored the combination and the other showed a negligible difference. Thus, the evidence does not support superior patient-assessed global improvement with combination therapy over FL monotherapy (Figure 4).

Risk of Bias (ROB)

**Figure 5:**
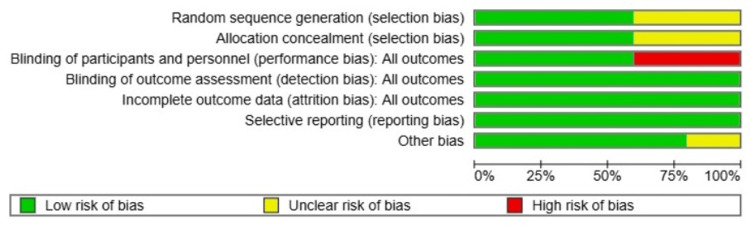
Risk of bias graph

While most domains across studies were judged as low risk, particularly for blinded outcome assessment, completeness of outcome data, and selective reporting, which were consistently rated low in all five studies, some concerns were identified. Two studies exhibited an unclear risk regarding random sequence generation and allocation concealment due to insufficient methodological description [8,9], and a high risk of performance bias was noted in two studies due to the inherent challenge of blinding participants and personnel in split-face laser trials. Despite these specific concerns, the universal implementation of blinded outcome assessment for objective scar evaluation supports the reliability of the primary efficacy data in this meta-analysis (Figure 5).

Discussion

Acne scarring is a widespread and troublesome complication following acne vulgaris, greatly affecting the quality of life of affected individuals. Atrophic scarring, marked by depressed scars on the skin surface, is a common sequela of the post-inflammatory process occurring following inflammatory acne breakouts. Although a host of treatment modalities, such as topical treatments, filler injections, chemical peels, and laser treatments, are current options, the best treatment strategy yielding optimal long-term results is yet under investigation [19]. The benefit of a combined treatment strategy integrating two promising treatment modalities: FMR, promoting the synthesis of collagen by inducing a host of minute punctures on the skin, and FL, improving existing scars by eliminating any cellular abnormality with a new, healthy skin response [20].

This meta-analysis brings together the latest collective evidence from five split-face randomized controlled clinical trials to determine the additive effectiveness of a combination approach using FMR alongside FL, a very common combination strategy currently in clinical practice for atrophic scarring in acne patients. The crucial result is that, in relation to three important effectiveness measures, FMR+FL fails to provide a significantly more advantageous outcome for the average patient than a stand-alone FL approach. The collective OR of 1.22 in terms of achieving a significant 50-75% reduction highlights this, suggesting that, for a variety of patients, a combination therapy approach, while adding high cost, time, and other contributory side effects, may not dramatically increase the chances of noticeable success [8,9].

Despite the lack of significance for the overall treatment effect when combined with both AFL and NAFL, the data set from the AFL group does deserve closer scrutiny regarding the suggestive borderline significance of the treatment effect. There may indeed be a certain specific synergistic effect due to the AFL causing channeled micro-injury with heavy coagulation necrosis in the dermis, potentially facilitating the deeper tissue penetration and heating profile of the radiofrequency treatment, hence potentiating a more profound collagen contraction and remodeling in the mid-to-deep dermis [4,5]. On the other hand, the NAFL only induces controlled dermal coagulation without any epidermal injury and therefore does not adequately potentiate the radiofrequency treatment effect, hence yielding only a modest but insignificant treatment effect [4,5]. Current paradigms of laser-tissue interactions for ablative injury actually propose a defined modulation of the wound-healing cascade by complementary and strategic utilization of other device technologies aimed at the optimization of fibroplasia and subsequent dermal reorganization [7,21].

In fact, analysis of physician-rated ECCA scores also does not show significance, with profoundly high, persistent heterogeneity (I^2^=91%). This high level of inconsistency suggests that the included studies are measuring treatment effects at different levels, most likely due to clinical heterogeneity and substantial variability in treatment parameters that are not solely attributable to laser technology. Some of these points of variation include scar morphology at base (e.g., predominance of rolling vs. icepick), Fitzpatrick skin type, depth and density of radiofrequency microneedles, laser parameters, and the number of sessions [5,22]. These specific divergent studies in NAFL add a prescient point about why particular technical approaches or a population of interest would radically affect published outcomes. Such clear delineation is a crucial counterpoint about why "FL" and "FMR" merely denote a range of technology, where specific operational details tend to be prime determinative factors in efficacy [6,7].

Regarding patient-reported global improvement, a lack of significant difference represents a critical outcome from a patient-centered care point of view. It suggests that, on average, patients do not feel a substantially superior aesthetic outcome with the combined approach when considering the possible increased degree of objective physical improvement as intimated by some AFL studies [15,23]. This obvious gap between objective measurement and subjective improvement is a long-observed phenomenon with esthetic dermatology and likely depends on variables including but not limited to pre-treatment expectations, a prioritization of scar texture over pigmentation or vice versa, and post-procedure recovery tolerance, among others [7]. The substantial degree of heterogeneity for this outcome, partly accounted for by the exclusion of one trial, also implies a set of complex variables, including but not limited to post-procedure pain and follow-up care, clinician-patient interaction, and possibly even coordinator-clinician. From a clinical point of view, the above finding recommends a personalized minimal approach in contrast to a universal strategy of a combination of treatments. For mild to moderate scarring with an established NAFL as the target platform, the initial approach of laser monotherapy would appear to be sound, deferring to a combination approach in the non-responders. For those selected with an AFL target, especially in the more severe or texturized areas of scarring, a sound argument can be proposed in initiating a combination approach, although this would be in the context of a signal detected in a small number of studies [5,7]. It would be necessary to consider this in the context of a potential increase in the risk of prolonged erythema, edema, and dyspigmentation in the context of the double approach of ablative and thermal modalities [6].

Limitations

This meta-analysis has several important limitations. First, the total number of included studies (n=5) and patients (n=116 patients) remains modest, limiting the statistical power to detect smaller but clinically relevant effects, particularly within subgroups. Second, the substantial and sometimes inexplicable heterogeneity for continuous variables (ECCA and patient satisfaction) undermines the certainty and generalizability of the aggregate analysis, though subgroup analysis could, to some extent, address these variables. Third, as meta-analyses are dependent on the outcome reporting in original studies, potentially underemphasized variables could also play crucial roles, such as durability beyond 12 months, qualitative aspects of scarring, and detailed analysis of adverse events. Finally, as in all procedural dermatology trials, blinding of physicians and patients is impossible, which could form the basis of performance bias, though the blinded assessment of outcome does reduce this concern in objective outcome variables.

## Conclusions

This meta-analysis evaluated the efficacy of FMR combined with AFL compared to AFL alone for treating post-acne atrophic scars. The pooled results showed no statistically significant differences between the two treatment modalities across the analyzed outcomes, including the achievement of 50-75% improvement in acne scars, changes in ECCA scores, and global improvement scale scores.

While FMR+AFL demonstrated a trend toward improved outcomes in some measures, the differences were not statistically significant, and high heterogeneity in certain outcomes limits the reliability of the findings. The consistently low ROB among the included studies supports the methodological quality of the evidence. However, the small sample size, high heterogeneity, and lack of significant results highlight the need for further high-quality randomized controlled trials with larger sample sizes and standardized outcome measures to better evaluate the potential benefits of combined FMR+AFL therapy for post-acne atrophic scars.

## Appendices

**Table 2:** Search terms

Search terms	Library	Number of retrieved articles		Number of articles	Causes for exclusion
(fractional microneedling radiofrequency) AND (fractional laser) AND (acne scars)	PubMed	44	Included after removal of duplications	191	Automatic duplicate removal
	Science direct	100	Included after other exclusions	105	Review articles
	WOS	38	included after abstract screening	7	-
	Scopus	33	included after full text screening i.e. to extraction	3	irrelevant (n=2), registered trial (n=1), duplicate (n=1)
	Central	20	-	-	-
	Manual	2	Final included	5	-
	Total	235	-	-	-
